# Integrated System Responses for Families Impacted by Violence: A Scoping Review

**DOI:** 10.5334/ijic.7542

**Published:** 2024-05-21

**Authors:** Claire Gear, Chien Ting, Carey Manuel, Elizabeth Eppel, Jane Koziol-McLain

**Affiliations:** 1 Centre for Interdisciplinary Trauma Research, School of Clinical Sciences, Auckland University of Technology, Private Bag 92006, Auckland 1142, New Zealand; 2 Āio Consulting Limited, Tauranga Moana, New Zealand; 3 School of Government, Victoria University of Wellington, Wellington, New Zealand; 4 Centre for Interdisciplinary Trauma Research, School of Clinical Sciences, Auckland University of Technology, Auckland, New Zealand

**Keywords:** system integration, service delivery, violence, health care, Indigenous, complexity theory

## Abstract

**Introduction:**

Violence within families is a complex problem which significantly impacts health and wellbeing. Despite the ubiquitous call for integrated family violence service delivery, integrated approaches vary significantly and challenges to implementation remain. This scoping review explored how integrated approaches to family violence service delivery are conceptualised within international and Aotearoa New Zealand literature.

**Methods:**

Following a documented scoping review process identified from literature, dynamic interplay between system agents within integrated family violence service delivery were mapped with the assistance of a complexity theory lens. We analysed characteristics of included studies, agents involved, how they interacted and the methods and mechanisms of integration among them.

**Results:**

Seventy-two published reports were included. The most common interactions occurred between statutory agencies such as police and child protection. While health care service providers were included within 55 studies, their engagement was often peripheral. Qualitative analysis elucidated three broad pathways to service delivery impact underpinned by systems-centred, person-centred, or Indigenous-centred worldviews.

**Discussion and Conclusion:**

Integrated approaches to family violence service delivery are highly variable. Despite a strong assumption that integration leads to improved safety, health, and wellbeing for care-seekers, most studies did not include evidence of such impact. Consideration of how worldviews characterise service provision provides insight into why integration has proven challenging over time.

## Introduction

Globally, family violence is recognised as a complex public health problem constituted by constantly evolving social, economic, health and cultural elements [1,2]. The gender-based nature of family violence disproportionately affects women and children, stemming from societally accepted gender inequalities and discrimination [3]. In Aotearoa New Zealand (NZ) (hereafter referred to as Aotearoa), population-based data estimates nearly two in three Aotearoa women, over two in three Indigenous Māori women, two in five Pacific women and one in three Asian women will experience a form of physical, sexual, psychological, controlling or economic violence by an intimate partner in their lifetime [4]. This data also evidences gender asymmetry with women experiencing more severe and frequent intimate partner violence than men [5]. Nevertheless, family violence does not discriminate, negatively impacting on all members of a family, including victim survivors, those who use violence and children [6]. Elder abuse is also a growing issue for Aotearoa, with one in ten New Zealanders over the age of 65 experiencing some form of elder abuse [7].

Internationally, integrated service delivery is a common health system policy and practice goal, yet evidence of consistent and improved care outcomes is yet to be proven. Lack of evidence is compounded by methodological challenges of measuring change in complex interventions across complex contexts [8,9]. Despite the aim of improved care, the focus of integration often lies with service delivery providers in reducing fragmentation and cost, with an assumption that this will lead to improved care [9,10,11]. Hughes, Shaw et al., [9] argue this contrasts with what matters to those accessing care, who value being seen, heard, and involved in service provision. Increasingly, service integration is being recognised as involving many complex dynamic relationships between macro, meso, and micro levels which shape care outcomes [8,9,10].

Recognising the intersecting determinants of violence within families, the World Health Organisation calls for a “holistic, integrated and coordinated response across different sectors, professional disciplines, and governmental, private and nongovernmental institutions” (p. 10) [3]. In Aotearoa, family violence systems have developed independently of one another over time, responsive to specific issues such as criminal justice, statutory child protection or health. This means families seeking help must navigate multiple compartmentalised systems, often while in crisis [1,3]. However, Aotearoa aspires to create an integrated approach to family violence service delivery that is more than just coordination between services, rather, all agencies and practitioners should have a collective understanding of family violence and the overall response system to respond effectively [1]. Most recently, the 25-year strategy, Te Aorerekura, set out six key shifts required to bring specialist, community and government sectors together to eliminate family and sexual violence [12]. Yet, despite repeated calls for integrated service delivery, generating care responsive to a multitude of justice, education, health and social needs, continues to be challenging at policy and practice levels [3].

Similar to Indigenous peoples globally, whānau (indigenous Māori collective) are disproportionately impacted by violence within whānau, with serious consequences for health and wellbeing. Violence within whānau stems from historical and colonial trauma, reinforced by systems and services that continue to perpetuate racism [13]. Understanding the layers of social entrapment is critical for disrupting these patterns of violence [14,15]. Western models of integration are predominantly framed around a particular problem or individual’s care, while kaupapa Māori methods (‘by Māori, for Māori’) address structural inequities that lead to poor health outcomes [16,17]. Responses are holistic, supporting whānau in self-determination of health and wellbeing grounded in cultural values [18,19]. Although the benefits of kaupapa Māori approaches are widely recognised, they are often marginalised by a requirement to validate outcomes from single government service perspectives (health, social or justice) using western evidence frameworks [16].

How to sustainably integrate family violence prevention and intervention initiatives remains challenging [1,20]. Internationally, participation of the health care sector in cross-agency work to reduce violence has been limited; and services addressing violence have not been well integrated into health systems [2,3]. In this study, we reviewed international scholarly and grey literature to explore how approaches to integrated family violence service delivery are conceptualised. We map how the agents involved interact with one another and how this shapes service delivery outcomes. While we aim to inform Aotearoa health care policy and practice, the findings will be of use to other countries developing integrated service delivery approaches to violence within families.

## Methodological Orientation

This study applies complexity theory (CT) as a theoretical lens to view the dynamic interplay between system elements shaping and been shaped by the responses to those impacted by violence [21]. Increasingly used in health care research, CT reconceptualises health care delivery systems as complex adaptive systems (CAS) [22,23]. In CAS, system ‘agents’ (an individual such as a nurse or care-seeker, or a collective such as an organisation and its codified and uncodified processes and routines) are constantly interacting and adapting to one another. The resulting co-evolution of system agents’ behaviour generates patterns of interaction that organically over time lead to new behaviours (self-organisation) and eventually the emergence of new system structures [23]. Our CT lens directs our focus towards identifying and extracting characteristics of interaction between agents that give rise to patterns of outcomes, such as dominant discourses, reinforced patterns and system behaviours that shape the understanding and application of an integrated approach to violence within families or whānau. This innovative theoretical approach to a scoping review has the potential to provide a rich understanding of system interactions, contextually relevant for different service delivery settings and populations.

## Methods

Scoping reviews include a range of types of evidence and are commonly used to map a literature landscape to inform policy and practice [24]. The research team developed and published a protocol [25] following the framework laid out by Arksey and O’Malley [26] and refined by Levac, Colquhoun et al., [27]. We chose this framework as it allows for qualitative data analysis [24], whereas other frameworks argue this is not within the remit of a scoping review [28]. In summary the approach proceeds in four sequential steps: 1) broad search based on terms generated from the research questions; 2) decisions on inclusion or exclusion based on abstract review against the inclusion criteria; 3) in-depth review of the included papers, and 4) qualitative discourse analysis of the content of the included studies. These steps are elaborated in the following sections. The study answered the research questions: How are integrated approaches to family and whānau violence portrayed in current literature? Further, if, and how are health system agents portrayed in the name of an integrated approach and if, and how are Indigenous perspectives portrayed? Our five-person team included expertise in complexity theory, Indigenous Māori health, public management, family violence and discourse methodology.

### Search strategy

The search strategy, inclusion and exclusion criteria, screening and extraction process were piloted in February 2021 with three databases (Medline; Informit Indigenous Collection; New Zealand Family Violence Clearinghouse library). Following the pilot, the search for literature was conducted in April 2021 following the participant, concept and context (PCC) [29] scoping review format (Table 1) with specified search terms (Table 2) and data sources (Table 3). Reference lists of all reports included for review were searched for additional reports [25]. Utilising Covidence (extraction 2.0) [30], two team members conducted data extraction, with consensus reached by a third reviewer, and disagreements resolved in wider team discussion. As the purpose of the scoping review was to map what exists in the current literature, no quality assessment tools were utilised in this review [24,28]. Inclusion and exclusion criteria were substantiated at regular team meetings.

**Table 1:** Scoping review inclusion and exclusion criteria.


	INCLUSION CRITERIA	EXCLUSION CRITERIA

**Participants**	System agents involved in responding to violence within families or whānau. ‘Agent(s)’ may be a collective such as a professional practice discipline (nurses or doctors), organisation or service (health or social).	Literature that does not discuss the interaction between at least two system agents that provide services.

**Concept**	Interaction between system agents responding to families and whānau impacted by violence.	Speculates on what integrated service design ‘ought to be’ but does not report actual service delivery interaction.

**Context**	System responses to families or whānau impacted by the family violence as defined in the protocol.	Literature related to violence occurring outside of familial relationships.

**Types of evidence**	Reviews (e.g., systematic, or narrative reviews), protocols for planned studies, peer-reviewed research articles, policy, strategy, or guidelines, full-text articles.	Reports published before 2010, Not written in English, Editorial articles, Abstracts or posters, Articles where full text is unavailable.


**Table 2:** Search terms.


KEYWORD		SEARCH TERMS

(1)	integrate	(“Integrated response” OR “integrated care” OR integration OR integrated OR inter-agency OR interagency OR cross-agency OR cross-sector OR multi-agency OR multi-sectorial OR collaboration OR joined-up OR cross-government OR network OR networked OR “system response” OR “comprehensive response” OR coordinated OR partnership) AND

(2)	family	(family OR whānau OR domestic OR children OR “intimate partner” OR tamariki OR interpersonal OR familial OR intrafamilial) AND

(3)	violence	(violence OR harm OR abuse OR “family violence” OR batter).

(4)	Indigenous	Indigenous, Māori, Māori-led, whānau-centred, whānau-based, “whānau first”, “Mana Wāhine”, “Mana tāne”, “whānau violence”, “kaupapa Māori”

(5)	New Zealand	‘*Zealand*’


**Table 3:** Study types and sources.


METHOD	TYPE	SOURCES

Database search	Published journal articles	CINAHL (via EBSCO), MEDLINE (via EBSCO), Cochrane Library (via OVID), PsycINFO, Scopus, Informit Indigenous Collection, NZ Family Violence Clearinghouse library

Manual search	Indigenous journals	MAI, Te Kaharoa

Google programmable search	Policies and grey literature (e.g., guidelines, strategy, and commissioned reports)	World Health Organisation, UN Women, VAWnet, Futures without Violence (U.S.A.), Australian institute of family studies, NZ Family Violence Death Review Committee, NZ Ministry of Social Development, NZ Oranga Tamariki Ministry for Children, NZ Joint Venture for Family Violence and Sexual Violence, NZ Ministry of Justice, NZ Police, NZ Ministry of Health, NZ Māori Reference Group for the Taskforce for Action on Violence within Families, NZ Office of the Commissioner for Children, E Tū Whanau, Domestic violence evidence project, Pacifica Proud


### Charting the data

Our analytical lens guided attention to agent interaction patterns self-organising into system behaviour. The data extraction table [25] was designed to draw out the definition and notion of integration presented by authors, focusing on examining what system agents were included, their relationships and how they were involved in an integrated approach. The design of the extraction table followed the PCC with an additional focus on Indigenous health equity. Over several analysis meetings four research team members applied a CT lens to extracted data and one researcher applied a Te Ao Māori (the Māori world) lens, examining characteristics of included literature (see Table 4) and methods and mechanisms of system agent interaction to identify patterns of interaction. In answering our primary research question, how are integrated family violence responses portrayed in literature, four themes were initially identified: (a) multidisciplinary responses (e.g., inter-agency meetings), (b) the notion of success (e.g., response or integration effectiveness), (c) a lack of specified pathway to impact and (d) Indigenous concepts and worldviews. We also noted integration mechanisms (e.g., safety plans, protocols, governance) and relational integration mechanisms (e.g., adaptability, willingness, commitment) and critiqued what was missing, variations, power relations and limitations of the scoping review. Informed by Schmidt’s [31] theorisation of the interactive relationship between power and ideas and the authors’ Triple R Pathway [32], a subsequent analysis meeting considered these themes across three levels: philosophy, policy and practice. At the philosophy level, we found four problem frames related to our research questions: (a) integrated responses offer safety for individuals, and accountability for service providers and users of violence (dominant problem frame), (b) system bifurcation favouring predominately social or justice responses allowing for marginalisation of health care, (c) multiple problem frames underpinning Indigenous responses ranging from being disconnected to Indigenous worlds, to Indigenous-led responses, and (d) lack of understanding of response success and/or impact due to non-inclusion of whānau and families voices. These value framings aligned with policy discourse and the logic of integration applied within the literature, leading to the identification of the three Pathways. The interdisciplinary nature of the research team Indigenous supported iterative and critical examination of the complexity and diversity involved within the integrated approaches described in the literature. As recommended by Levac, Colquhoun et al., [27], the research team presented preliminary findings to seven frontline service providers from justice, social, health and kaupapa Māori health to generate collective sensemaking on whether the scoping review findings resonated within their ‘real-world’ of practice.

**Table 4:** Characteristics of reports (N = 72).


Study types	Original research	21

	Report	19

	Programme evaluation	15

	Meta-synthesis	11

	Other (guidelines, framework, strategy)	6

Countries of origin	Australia	30

	USA	16

	UK	12

	New Zealand	11

	Australia & USA	1

	Australia & UK	1

	Canada	1

Focus on types of violence addressed	Family violence (multiple)*	55

	Child abuse and neglect	12

	Intimate partner violence	4

	Elder abuse	1

System agents represented**	Health	55

	Justice (courts, correction)	45

	Child protective agencies	45

	Police	43

	Community and NGOs (Men’s services, counselling, advocacy)	40

	DV specialist	35

	Social services	31

	Housing	17

	Education	14

	Drug and Alcohol	8

	Disability	4

	Finance	1

Theoretical position	Specified	41

	Not specified	31


*Reports including multiple forms of violence including physical, mental, sexual, coercive control, financial, violence against women and their children, children living with violence, child physical, mental, and sexual abuse, sibling sexual abuse, violence against Indigenous women, whānau violence, honour-based violence, elder abuse, and women using force. **Inclusion criteria required a minimum of two agencies involved in the integrated service, therefore n > 72. We have maintained the terms used by authors of the selected reports, it is likely that *Community and NGO services* overlaps with *DV specialist* services.

## Findings

**Figure 1:**
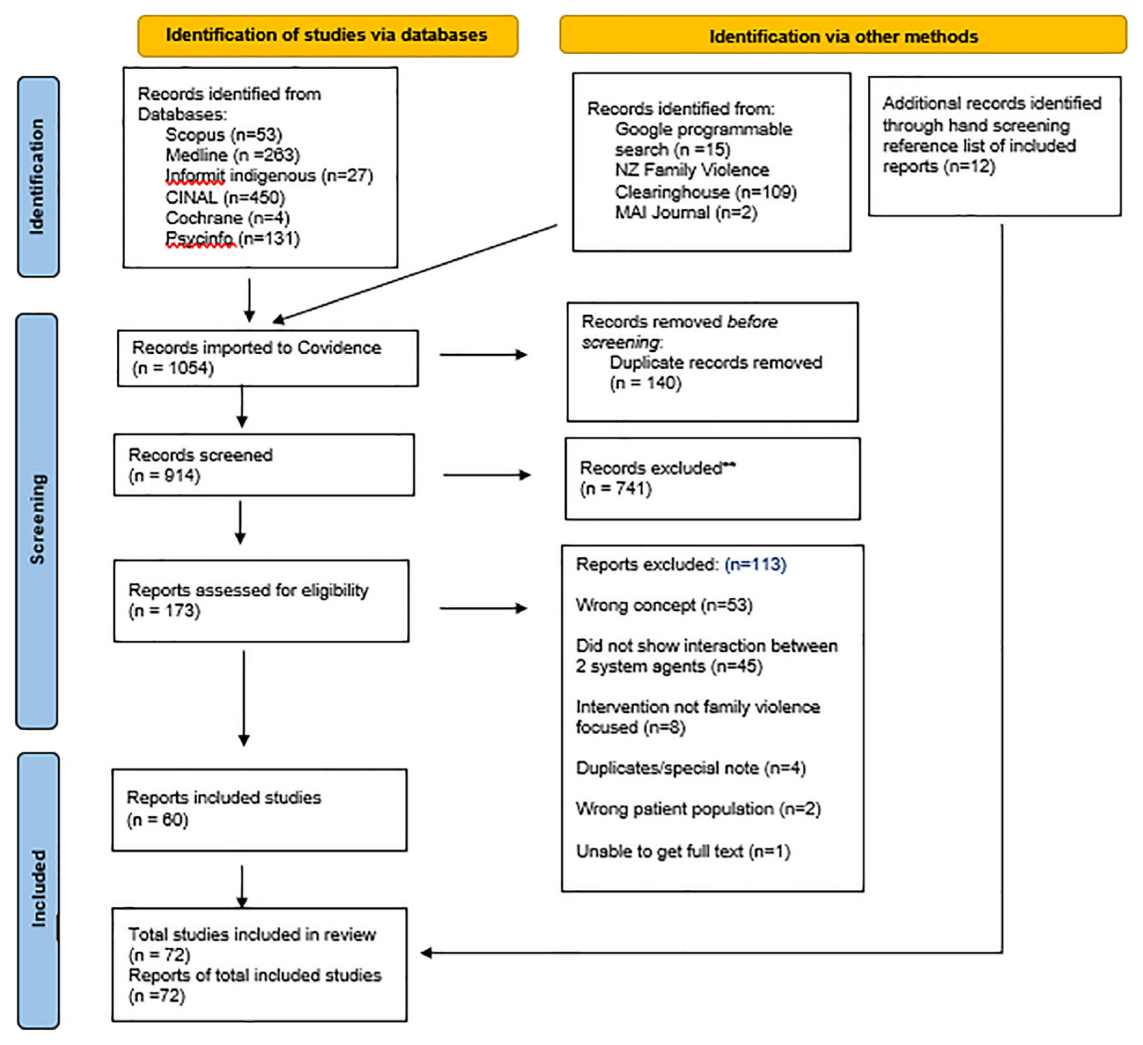
Database search results.

From a total of 1054 records, 72 reports were selected for inclusion (see Supplement for list of included studies). The screening process, reasons for exclusion and numbers of included texts are presented in the PRISMA-ScR [33] flow diagram (Figure 1). Common reasons for exclusion included: (a) no explicit interaction between at least two system agents, (b) speculates on what integrated service design ‘ought to be’ but does not report actual service delivery interaction, (c) studies that discuss co-occurrence of issues (e.g., HIV and intimate partner violence) rather than service delivery integration.

### Study characteristics

The general characteristics of reports incorporating an integrated approach to family violence service delivery are outlined in Table 4.

### System agents involved in an integrated approach to family violence service delivery

All system agents represented in the reported integrated approach to family violence were extracted. While health care service providers were often mentioned (n = 55), there was little detail regarding how health providers interacted with other service providers aside from perfunctory mechanisms, such as a memorandum of understanding or referral. For example, while information may be shared with a health provider, what follow up action was taken was not reported. In many cases, health services were mentioned as a potential referral agency, without any follow-up mechanism or feedback loop identified. Health agents included mental health, general practice, maternity care, home health nursing, telehealth, and local health providers. The most common interactions found occurred between statutory agencies such as police and child protection. Where health agents were involved, it was common for police or justice to also be involved (90%) or child protection services (50%) and in the leading role.

### Aims, methods and mechanisms of integration

Methods and extent of integration was variable across all reports. Generally, the logic behind integration was not explicit, making it difficult to identify how the approach would lead to improved safety, health, and wellbeing. Theoretical positioning was identified within 41 reports including for example appreciative inquiry, co-design, family-centred and Indigenous-based approaches. Methods and mechanisms to integrate service delivery commonly included multi-disciplinary meetings, service provider notifications, database sharing and mandatory reporting. Information sharing was the common method to integrate services, with mechanisms varying in intensity, ranging for example from utilising shared databases or email [34,35], to face-to-face meetings to discuss client needs [36]. Other methods of integration included co-location of services (on-site advisory), cross-agency training, case-coordination, development of shared safety plan, referral, and follow-up. Few reports, however, explored ‘how’ agents translated and negotiated different needs in interaction with one another. In one example of addressing ‘how’, authors reported the relational aspects considered necessary to build integration such as willingness, commitment, and atmosphere of mutual respect. They also recognised barriers to integration such as tensions between organisational power, practices or objectives [35].

## Integrated family violence service delivery: pathways to impact

Analysis of extracted data, using complexity and Te Ao Māori lenses, found integrated approaches to family violence service delivery cluster around three broad pathways (see Table 5). These ‘Pathways to Impact’ are a product of interactions occurring between particulars ystem agents and their contexts. The way the interactions are framed reveals a logic about how family violence is viewed, the associated values that shape what is considered important when responding and the labels that are used (e.g., victim, service user, whānau). In turn, this framing creates a path dependency for how service providers view service delivery, including how integration is spoken about in policy and practice (discourse), the approach implemented to integrate services (the inferred integration logic), and the possible impact service delivery may have for those seeking care for family violence (impact). We theorise these pathways as dynamic, that system agents move across and negotiate pathways depending on their context, relationships, and knowledge needs within different moments in time [37]. For example, interagency team meetings bring together multiple service providers and worldviews that interact, influencing the pathway to impact.

**Table 5:** Integrated Family Violence Service Delivery: Pathway to Impact.


	WORLDVIEW	PRACTICE DISCOURSE	INTEGRATION LOGIC	IMPACT

	A way of looking at the problem that shapes what is considered important	How integrated family violence service delivery is spoken about in policy and practice	The approach taken to integrate services in policy and practice	The possible impact integrated family violence service delivery may have for care-seekers

*Systems-centred*	Focuses on service delivery agents and mechanisms of interaction between them	Safety and accountability via information sharing	Prescribed processes e.g., protocols, mandates, interagency meetings	System efficiency

*Person-centred*	Focuses on individual, family or whānau service delivery	Individual assessment and care	Symptom and needs-based report & referral	System outputs and outcomes, known or unknown

*Indigenous-centred*	Focuses on inherent, seen or unseen, relationships between person, family or whānau, culture and values	Connectedness, wellbeing and balance	Connection to metaphysical elements and incorporation of these in ordinary tasks	Hauora and healing


The three broad pathways of integrated family violence service delivery found within the literature we labelled (a) Systems-centred, (b) Person-centred, and (c) Indigenous-centred (see Table 5). It was evident that each pathway reflected a distinctive worldview shaped by the *Discourses of Practice*, or the way integrated family violence service delivery is referred to. It underpinned the *Integration Logic* applied and set a trajectory toward possible *Impact*. These three distinctive worldviews generate system boundaries around parts of the whole system response to family violence reflective within policy and practice [38]. For example, police focus on the immediate stopping of violence, while health professionals address the symptoms or injuries from violence. While some system agents (individually and collectively) did tend to reflect a particular pathway, this was dynamic and dependent on context. It may even be prescribed, for example health professionals being legally required to report child abuse and neglect. It is important to note that across the literature we examined, there was a missed opportunity for *Impact* to generate a feedback loop to critique the integrated approach to service delivery, including recognition of the *Worldview, Discourse of Practice* and *Integration Logic* in use.

### System-centred pathways to impact

We identified an organisational Systems-centred *worldview* that focuses on the providers involved in service delivery and the mechanisms of interaction between them. This worldview generates a *discourse of practice* on establishing safety for families and whānau, and accountability for users of violence. Family violence service providers often used their institutional power to manage ‘cases’ through standardised processes such as risk assessment, safety planning and action agreements, thereby removing the autonomy of care-seekers in making their own choices in receiving care. This systems-centred *integration logic* focuses on information sharing via mechanisms between system agents. These are often in the form of referrals and interagency meetings that delegate tasks each service provider needs to undertake as part of their role within the team. Systems-centred pathways involve directing responses to crisis events which trigger prescribed protocols, service mandates and agreements. High-risk cases were usually prioritised for action, with little support for low-risk cases and long-term healing. Very few reports elaborated on how these mechanisms of integration enabled agents to work better together. For example, Stanley, Miller et al., [34] noted the limitations of police family violence notifications to social workers omitting information about children’s exposure to violence. Systems-centred *Impact* is directed toward system efficiency, that is, what service providers need to do to play their part, and how they may best work together. The voices of care-seekers were rarely included in decision-making or response evaluation. Agents working within a Systems-centred pathway commonly included police, child protection, case managers, advocates, and health professionals.

### Person-centred pathways to impact

A Person-centred *worldview* focuses on service provision to the individual, family or whānau seeking care at a particular point in time. *Discourses of practice* informed by a Person-centred worldview focussed on individual assessment and care for the person presenting to a service provider. Practice involves a transition of power by the provider facilitating care needs and referral to other services, informed by their understanding of the profession they worked within. Person-centred *integration logic* focuses on individualised needs and symptom-based report and referral based on understanding at the time of consultation, with follow-up care undertaken dependent upon the individual agency of provider and client. Person-centred *Impact* focuses on system outputs (e.g., number of referrals) and outcomes (e.g., health outcomes) often shaped by funding models. While outputs are often quantifiable, outcomes may or may not be known, for example, whether a referral is acted upon by the client or used by the provider. Agents in this worldview commonly included general practitioners, schools, local domestic violence agencies, or community service providers.

### Indigenous-centred pathways to impact

An Indigenous-centred *worldview* focuses on holistic service delivery embedded in the cultural context through recognition of the inherent, seen or unseen, relationships between a person, their family and whānau, culture, values, health, and healing. For example, Indigenous-centred approaches recognise the range of kinship relations and interconnectedness within and between families and the Indigenous traditions that guide family interactions [39,40,41] and include recognition of the impacts of colonisation, racism, and collective trauma over time [41,42,43]. We found very few Indigenous-centred approaches to service delivery within the literature. Further, approaches that were Indigenous-centred, did not operate independently of wider non-Indigenous service providers and were required to be responsive to other worldviews, practice discourses and integration logic, as they also impact Indigenous peoples. This was an obligation not required of non-Indigenous providers. Agents ascribing to this worldview commonly included Indigenous providers [44] with a delivery philosophy that is localised and inclusive. Indigenous-centred *discourses of practice* inherently differed from Systems-centred and Person-centred pathways in the conceptual understanding of family violence, acknowledging a wider range of culturally important kinship relations, violent behaviours, and understanding of collective trauma [36,41,44]. They also held a different understanding of what constitutes an ‘integrated’ response, focusing on “prevention and integration within cultural health and healing families”, rather than service provider integration and efficiency [36]. Indigenous approaches offered appreciation of the sources of family violence as well as a relational, multi-generational long-term approach to prevention. While notions of power within literature promoting an Indigenous-centred worldview were not always explicit, we theorise power becomes a ‘continuous multi-directional flow’ among the system elements. *Integration logic* shaped by an Indigenous-centred worldview promotes connection to meta-physical elements and the incorporation of these within ordinary tasks. This occurs from the bottom up, with ‘place-based’ responses owned and managed by Indigenous peoples for specific contexts and populations [44]. The logic recognises interdependencies, cumulative effects of needs, and the importance of timing and sequencing of actions [36,44]. Indigenous-centred *impact* focuses on long-term hauora (wellbeing) and healing through multiple and ongoing connections to meaningful aspects to families and whānau. We found Indigenous-centred pathways were often marginalised by the dominance of Systems-centred and to a lesser extent the Person-centred pathways. Within these pathways, *discourses of practice* regarding Indigenous populations ranged from being ‘othered’ or ‘problematic’ with selective or no rationale for inclusion [39,40,45,46,47,48,49], to inclusion for cultural sensitivity [41,42,50,51,52], to Indigenous-led approaches [36,44,53]. This range of responsiveness means Indigenous peoples are recognised across the three Pathways to Impact, even if the response has minimal outcome effectiveness.

### Consultation: Whakawhitiwhiti Kōrero (Discussion)

We presented the preliminary findings of the scoping review to seven frontline family violence service professionals (including Māori and non-Māori). Through discussion we found they were unsurprised by the findings of the scoping review. They noted the importance of listening and acting on the voices and experiences of care-seekers to improve understanding of how services provided help, noting that providers themselves had difficulty in capturing this information to improve policy and practice. All providers considered building relationships among service providers and with whānau and families necessary to success. Even over significant periods of time, they find relationships continue to be fraught with difficulties and the question ‘why are they not working’ has not been asked. Providers expressed frustration at the way colonising generalist service framework models tended to fragment service user perspectives and constrained potential service delivery addressing long-term support for multigenerational healing and restoration.

## Discussion

Integrated service delivery has long been advocated to improve outcomes for those impacted by family violence. This scoping review explored how integrated family violence service delivery is portrayed within international and Aotearoa literature, with a specific focus on the role of health care and Indigenous-centred care within integrated service delivery. Our findings call attention to how particular worldviews of integrated family violence service delivery shape the perception of the problem for different system agents. These worldviews strongly shape the type of response considered, constrain the possible range of solutions under consideration and become legitimised within policy and practice [54,55,56]. Connecting back to how these worldviews lead to different characteristics of service provision provides insight into why integration has proven challenging [37,38]. The three Pathways to Impact found in the literature represent an attempt at differentiating between the worldviews of different system agents involved in integrated family violence service delivery. The three pathways have implications for service provision and provide a theoretical way to reduce the complexity involved. In the real-world of practice, however, system agents move across and negotiate pathways depending on their context, relationships, and knowledge needs within different moments in time [37].

*How are integrated approaches to family and whānau violence portrayed in current literature?*

Findings of this scoping review strongly align with literature on health care integration generally, with high variation, a focus on service provider integration and little evidence of effectiveness [8,9]. Diversity of integration models has usefully been conceptualised by other scholars on a continuum, allowing for a nuanced view of how integration works across different contexts [41,57]. Our analytical lens provides further nuance by presenting a nonlinear view of how integration is shaped depending on circumstances and contexts in practice. The complexity of family violence adds to the growing awareness of the number of dynamic interactions occurring within micro, meso, and macro levels for individuals, collectives and service providers, and the consequent difficulty in measuring change [10,11]. We found approaches to integrate family violence service delivery are highly variable within the literature, with little reported on why or how integration occurs, beyond perfunctory mechanisms. There is a strong assumption that integrated approaches to family violence lead to improved safety, health, and wellbeing for care-seekers, yet the majority of reports did not include evidence of such impact. Measures used to explore impact were often short term and inadequate to demonstrate change.

A complexity theory lens helps to view the Pathways to Impact as socially constructed system boundaries. Within a complex system, boundaries do not create separation between system parts, rather they constitute what is bounded. As system agents interact, ways of looking at the problem and associated values become bounded together, shaping what emerges from that part of the system [37,58]. For example, the systems-centred pathway focuses on service delivery agents and mechanisms of interaction between them, generating value on system-efficiency outputs. Understanding the history of system interaction provides insight into how boundaries have developed and been maintained [56]. Boundaries also provide insight into how other ways of looking at the problem become marginalised, as they challenge the dominant problem boundaries [37]. Understanding the Pathway to Impact other service providers operate within could help to strengthen relationships and ways of working together, leading to a wider understanding of impact. Yet such mechanisms are currently unclear and unevidenced, despite recognition of the potential benefits working together could have for service providers and care-seekers [59]. Complexity theory offers the concept of ‘boundary objects’ as mechanism(s) for collaboration and knowledge translation between different agents. A boundary object is a shared thing or concept that opens communication across professional divides. Although it maintains a recognisable form, it has interpretative flexibility or multiple meanings for different groups [60]. For example, Fleming, Safaeinili et al., [11] critique the use of ‘high-risk patients’ as a shared intervention concept across health care and social service providers. The analytical lens of this scoping review calls attention to how agents interact across the Pathways depending on need. Given these interactions, we suggest there may be unseen boundary objects working, or not working, that could be harnessed to improve service delivery integration.

*If, and how are health system agents portrayed in the name of an integrated approach?*

We theorise health system agents ascribe to a Person-centred pathway of service delivery. While the count of health system agents included in integrated family violence service delivery was high, little was reported on how health agents interact with other service providers aside from perfunctory standardised mechanisms that leave system boundaries intact and rely on providers to adapt for use. Health system agents tended to operate on the periphery of integrated family violence service delivery and were often either a point of entry to family violence service provision, by referring onto other services, or referred into by family violence service providers. We theorise health system agents tend to integrate with other health services, rather than with family violence service providers outside health. Person-centred pathways focus on the individual, family or whānau seeking care, potentially marginalising understanding of social, political, cultural, and structural factors involved [61]. Recognising family violence as a key determinant of ill-health requires an understanding of the context violence occurs within [32]. Without this context, understanding of the role the health system can play in responding to family violence may be constrained.

*If, and how are Indigenous perspectives portrayed?*

Indigenous-centred pathways represented a conceptually different understanding of both family violence and the notion of integration that conflict with Systems-centred and Person-centred Pathways to Impact. Systems-centred and Person-centred pathways often included an Indigenous component within integrated family violence service delivery. Indigenous components were generally designed to (a) provide Indigenous responses to Indigenous peoples and families; (b) safely guide Indigenous peoples and families through non-Indigenous systems and services, and (c) educate and upskill non-Indigenous providers to safely interact with Indigenous peoples and families. Indigenous peoples were often ‘othered’ within service delivery, added to as an ‘addition’, ‘variation’, or ‘alternative’ approach with the aim of providing culturally ‘safe’ or ‘responsive’ care. We question whether our search strategy limited the inclusion of Indigenous approaches as conceptual differences of ‘integration’ means our search terms may not be used by Indigenous peoples, focusing less on integration of services and more on care-seekers themselves. Similarly conceptual differences of ‘family violence’ may have limited inclusion of kaupapa Māori approaches that respond to family violence through addressing structural inequities. The Westernised concept of system integration tend toward a series of hub and spoke networks with the family at the extremity whereas an Indigenous-centred approach centres whānau and families with the network of service providers responding as needed.

*Gaps within literature*. Within the literature reviewed, there was little critique elucidating and testing the Pathway to Impact, i.e., whether the integrated approach led to whānau and families living free from violence. The voices of those seeking care were rarely included within reports, missing opportunities to understand experiences of service delivery and not repeat harmful responses, particularly important for Indigenous peoples for whom negative system narratives and inequities perpetuate trauma [62]. There was little response included for prevention, ‘low risk cases’ or ongoing care beyond crisis intervention, representing clear service needs. Literature often reported on what mechanisms of interaction were utilised, without elucidating how these mechanisms worked and what feedback loops were incorporated. Further gaps included integration with the education sector and service delivery within rural, remote, and low-income areas.

### Limitations

In designing this review, we aimed to source family violence interventions and services that generated interaction between at least two system agents [25]. The scope was therefore large, including a wide range of terms associated with integration, types of violence, and reports that discussed interaction between at least two system agents. The review focused on literature that discussed what was taking place, rather than guidance or recommendations on what should occur [25]. This criterion generated a wide range of approaches to integrated service delivery, while also limiting our search in other ways. For example, models of integration that addressed family violence as one issue within a wider range of health and social care issues, may not have been captured, raising the possibility that ‘true’ integration means interaction between agents are ‘unseen’ in service delivery.

The use of complexity theory applied to the scoping review process presented methodological challenges and benefits. Complexity theory seeks to identify patterns of system behaviour. In contrast, the scoping review process breaks down data into discrete ‘parts’, preventing a view of the ‘whole’. The chunks of data extracted become divorced from their context, preventing an understanding of relationships between system parts. Similarly, the nature of family violence is complex and to extract de-contextualised information out of a text does not appreciate the nature of this multi-layered subject. Nonetheless, use of complexity theory enabled a richer qualitative understanding of the quantitative findings to tell the story behind the numbers.

### Recommendations and future research

Designing integrated approaches to family violence service delivery requires a clear focus on the Pathway to Impact, including a continuous feedback loop to assess trajectory of change, including unintended consequences. Future research examining the impact of integrated family violence service delivery is needed, with critical reflection on the background ideas and assumptions of the Pathway taken. Indigenous-centred approaches provide opportunity to move away from a focus on acute, high-risk responses, account for how histories of displacement and disruption shape intergenerational trauma and to value the importance of centring local culture and history in recovery and healing. Recognising the complexity involved in family violence suggests use of complexity theory is a strength in exploring the dynamic nature of integrated family violence service delivery.

## Conclusion

Integrated family violence service delivery is widely advocated to improve outcomes for those seeking care for family violence. This scoping review found approaches to integrated service delivery are highly variable with little assessment of improved outcomes. We identified three broad Pathways to Impact that provide insight into how different approaches to service delivery are enacted. Critical reflection on how service providers move across these Pathways in practice can help to identify strengths and weaknesses in service delivery, working toward better unification in improving outcomes for care-seekers.

## Additional File

The additional file for this article can be found as follows:

10.5334/ijic.7542.s1 Supplemental File 1. Included reports (N = 72).
