# Supplementary material for: Integrated System Responses for Families Impacted by Violence: A Scoping Review
Source: Int J Integr Care. 2024 May 21;24(2):17. doi: 10.5334/ijic.7542 (PMC11122700; doi:10.5334/ijic.7542)
Supplement: Supplemental File 1. — Included reports (N = 72). [file ijic-24-2-7542-s1.pdf]

Supplemental File 1: Included reports (N=72)

Appleton-Dyer, S., & Soupen, A. (2017). *Rapid review of the Waitemata Safeguarding Adults from Abuse (SAFA) pilot: Report for the Waitemata Police*. Retrieved from Auckland, NZ https://nzfvc.org.nz/sites/nzfvc.org.nz/files/Synergia-final-report-of-the-SAFA-Pilot-5-April-2017.pdf

Australia's National Research Organisation for Women's Safety. (2020). *Working across sectors to meet the needs of clients experiencing domestic and family violence: Research synthesis*. Retrieved from NSW, Australia https://www.anrows.org.au/publication/working-across-sectors-to-meet-the-needs-of-clients-experiencing-domestic-and-family-violence/

Backhouse, C., & Toivonen, C. (2018). *National Risk Assessment Principles for domestic and family violence: Companion resource. A summary of the evidence-base supporting the development and implementation of the National Risk Assessment Principles for domestic and family violence*. Retrieved from Sydney, NSW: https://www.anrows.org.au/research-program/national-risk-assessment-principles/

Blagg, H., Williams, E., Cummings, E., Hovane, V., Torres, M., & Woodley, K. N. (2018). *Innovative models in addressing violence against Indigenous women: Final report*. Retrieved from Sydney, NSW: https://anrowsdev.wpenginepowered.com/wp-content/uploads/2019/02/4.3-Blagg-Final-Report.pdf

Blagg, H., Williams, E., Cummings, E., Hovane, V., Torres, M., & Woodley, K. N. (2018). *Innovative models in addressing violence against Indigenous women: Key findings and future directions*. Retrieved from Sydney, NSW: https://anrowsdev.wpenginepowered.com/wp-content/uploads/2019/02/4.3-Blagg-Key-Findings-and-Future-Directions.pdf

Breckenridge, J., Rees, S., Valentine, K., & Murray, S. (2015). *Meta-evaluation of existing interagency partnerships, collaboration, coordination and/or integrated interventions and service responses to violence against women: State of knowledge paper*. Retrieved from Sydney, Australia: https://anrowsdev.wpenginepowered.com/wp-content/uploads/2019/02/Integrated-Responses-Meta-Evaluation-Landscapes-State-of-knowledge-Issue-Eleven-1.pdf

Breckenridge, J., Rees, S., Valentine, K., & Murray, S. (2016). *Meta-evaluation of existing interagency partnerships, collaboration, coordination and/or integrated interventions and service responses to violence against women: Final report*. Retrieved from Sydney, NSW: https://anrowsdev.wpenginepowered.com/wp-content/uploads/2019/02/H4_3.2_IRME_WEB-270616-1.pdf

Carswell, S., Atkin, S., Wilde, V., Lennan, M., & Kalapu, L. (2010). *Evaluation of the Family Violence Interagency Response System (FVIARS): Summary of findings*. Retrieved from Wellington, New Zealand: https://www.msd.govt.nz/about-msd-and-our-work/publications-resources/evaluation/family-violence-interagency-response-system/index.html

Chung, D., Upton-Davis, K., Cordier, R., Campbell, E., Wong, T., Salter, M., . . . Bissett, T. (2020). *Improving accountability: The role of perpetrator intervention systems.* Retrieved from Sydney, NSW: https://anrowsdev.wpenginepowered.com/wp-content/uploads/2020/06/Chung-RR-Improved-Accountability.pdf

Clarke, A., & Wydall, S. (2013). 'Making Safe': A Coordinated Community Response to Empowering Victims and Tackling Perpetrators of Domestic Violence. *Social Policy and Society, 12*(3), 393-406. doi:10.1017/S147474641200070X

Cleek, E. A., Johnson, N. L., & Sheets, L. K. (2019). Interdisciplinary collaboration needed in obtaining high-quality medical information in child abuse investigations. *Child Abuse and Neglect, 92*(3), 167-178. doi:10.1016/j.chiabu.2019.02.012

Cowley, L. E., Maguire, S., Farewell, D. M., Quinn-Scoggins, H. D., Flynn, M. O., & Kemp, A. M. (2018). Factors influencing child protection professionals' decision-making and multidisciplinary collaboration in suspected abusive head trauma cases: A qualitative study. *Child Abuse and Neglect, 82*, 178-191. doi:10.1016/j.chiabu.2018.06.009

Cripps, K., & Habibis, D. (2019). *Improving housing and service responses to domestic and family violence for Indigenous individuals and families*. Retrieved from Melbourne, Victoria: https://www.ahuri.edu.au/research/final-reports/320

Diemer, K., Humphreys, C., Laming, C., & Smith, J. (2013). Researching collaborative processes in domestic violence perpetrator programs: Benchmarking for situation improvement. *Journal of Social Work, 15*(1), 65-86. doi:10.1177/1468017313504682

Domoney, J., Fulton, E., Stanley, N., McIntyre, A., Heslin, M., Byford, S., . . . Trevillion, K. (2019). For Baby's Sake: Intervention Development and Evaluation Design of a Whole-Family Perinatal Intervention to Break the Cycle of Domestic Abuse. *Journal of Family Violence, 34*(6), 539-551. doi:10.1007/s10896-019-00037-3

Dyer, C. B., Halphen, J. M., Lee, J., Flores, R. J., Booker, J. G., Reilley, B., & Burnett, J. (2020). Stemming the Tide of Elder Mistreatment: A Medical School-State Agency Collaborative. *Academic medicine: Journal of the Association of American Medical Colleges, 95*(4), 540-545. doi:10.1097/ACM.0000000000003028

Dyson, S., Frawley, P., & Robinson, S. (2017). *Whatever it takes: Access for women with disabilities to domestic and family violence services: Final report*. Retrieved from Sydney, NSW: https://anrowsdev.wpenginepowered.com/wp-content/uploads/2019/02/Disability_Horizons_FINAL-1.pdf

Family Law Council. (2016). *Family Law Council report to the Attorney-General on families with complex needs and the intersection of the Family Law and child protection systems: Final report - June 2016 (Terms 3, 4 & 5)*. Retrieved from Australia: https://www.ag.gov.au/sites/default/files/2020-03/Family-with-Complex-Needs-Intersection-of-Family-Law-and-Child-Protection-Systems-Final-Report-Terms-3-4-5.PDF

Family Violence Death Review Committee. (2016). *Family Violence Death Review Committee: Fifth report: January 2014 to December 2015*. Retrieved from Wellington, New Zealand: https://www.hqsc.govt.nz/assets/Our-work/Mortality-review-committee/FVDRC/Publications-resources/FVDRC-5th-report-Feb-2016-v2.pdf

Forsdike, K., Humphreys, C., Diemer, K., Ross, S., Gyorki, L., Maher, H., . . . Hegarty, K. (2018). An Australian hospital's training program and referral pathway within a multi-disciplinary health-justice partnership addressing family violence. *Australian and New Zealand Journal of Public Health, 42*(3), 284-290. doi:10.1111/1753-6405.12743

Frere, M. (2012). *A whole-of-government approach to family violence reform: A paper presented at the Family Violence Symposium*. Retrieved from Wellington, NZ: https://nzfvc.org.nz/sites/default/files/%27Whole%20of%20government%27%20approach%20to%20family%20violence%20reforms.pdf

Gmelin, T., Raible, C. A., Dick, R., Kukke, S., & Miller, E. (2018). Integrating Reproductive Health Services into Intimate Partner and Sexual Violence Victim Service Programs. *Violence against Women, 24*(13), 1557-1569. doi:10.1177/1077801217741992

Graves, K. N., Ward, M., Crotts, D. K., & Pitts, W. (2019). The Greensboro Child Response Initiative: A Trauma-Informed, Mental Health–Law Enforcement Model for Children Exposed to Violence. *Journal of Aggression, Maltreatment & Trauma, 28*(5), 526-544. doi:10.1080/10926771.2018.1490843

Gray, M. J., Hassija, C. M., Jaconis, M., Barrett, C., Zheng, P., Steinmetz, S., & James, T. (2015). Provision of Evidence-Based Therapies to Rural Survivors of Domestic Violence and Sexual Assault via Telehealth: Treatment Outcomes and Clinical Training Benefits. *Training & Education in Professional Psychology, 9*(3), 235-241. doi:10.1037/tep0000083

Gregory, A., Ramsay, J., Agnew-Davies, R., Baird, K., Devine, A., Dunne, D., . . . Feder, G. (2010). Primary care identification and referral to improve safety of women experiencing domestic violence (IRIS): Protocol for a pragmatic cluster randomised controlled trial. *BMC Public Health, 10*. doi:10.1186/1471-2458-10-54

Gregory, R., Green, R., & Brandenburg, M. (2010). Building relationships: An example of integrating family violence programs which support women and children. *Women Against Violence: An Australian Feminist Journal* (22), 41-49.

Hebert, S., Bor, W., Swenson, C. C., & Boyle, C. (2014). Improving collaboration: A qualitative assessment of interagency collaboration between a pilot Multisystemic Therapy Child Abuse and Neglect (MST-CAN) program and a child protection team. *Australasian Psychiatry, 22*(4), 370-373. doi:10.1177/1039856214539572

Herbert, J., Ghan, N., Salveron, M., & Walsh, W. (2021). Possible Factors Supporting Cross-Agency Collaboration in Child Abuse Cases: A Scoping Review. *J Child Sex Abuse, 30*(2), 167-191. doi:10.1080/10538712.2020.1856994

Herbert, J. L., & Bromfield, L. (2017). *Components of effective cross-agency responses to child abuse: A report for the NSW Ombudsman's Office (Report 2 of 2)*. Retrieved from Adelaide, SA: https://regroup-production.s3.amazonaws.com/documents/ReviewReference/324483034/components-of-effective-cross-agency-responses-to-abuse.pdf?response-content-type=application%2Fpdf&X-Amz-Algorithm=AWS4-HMAC-SHA256&X-Amz-Credential=AKIAYSFKCAWYQ4D5IUHG%2F20221208%2Fus-east-1%2Fs3%2Faws4_request&X-Amz-Date=20221208T011141Z&X-Amz-Expires=604800&X-Amz-SignedHeaders=host&X-Amz-Signature=b288e26bffb9ae01dde8c3058ac0c78ff66765841194bd5bf364170fab0a3f7e

Herbert, J. L., & Bromfield, L. (2017). *National comparison of cross-agency practice in investigating and responding to severe child abuse: A report to the NSW Ombudsman's Office (Report 1 of 2)*. Retrieved from Adelaide, SA https://unisa.edu.au/siteassets/episerver-6-files/global/eass/research/accp/national-comparison-of-cross-agency-practice-in-investigating-and-responding-to-severe-child-abuse-.pdf

Herbert, J. L., & Bromfield, L. (2017). *National comparison of cross-agency practice in investigating and responding to severe child abuse: CFCA Paper 47*. Retrieved from Melbourne, Australia: https://aifs.gov.au/sites/default/files/publication-documents/47_national_comparison_of_cross-agency_practice_in_investigating_and_responding_to_severe_child_abuse_0.pdf

Herbert, J. L., & Bromfield, L. (2019). Better Together? A Review of Evidence for Multi-Disciplinary Teams Responding to Physical and Sexual Child Abuse. *Trauma Violence Abuse, 20*(2), 214-228. doi:10.1177/1524838017697268

Huebner, R. A., Young, N. K., Hall, M. T., Posze, L., & Willauer, T. (2017). Serving families with child maltreatment and substance use disorders: A decade of learning. *Journal of Family Social Work, 20*(4), 288-305. doi:10.1080/10522158.2017.1348110

Humphreys, C., & Healey, L. (2017). *PAThways and Research in Collaborative Inter-Agency working: Collaborative work across the child protection and specialist domestic and family violence interface: The PATRICIA Project. Final report*. Retrieved from Sydney, NSW: https://www.anrows.org.au/publication/pathways-and-research-into-collaborative-inter-agency-practice-collaborative-work-across-the-child-protection-and-specialist-domestic-and-family-violence-interface-the-patricia-project-final-report/

Humphreys, C., Healey, L., Kirkwood, D., & Nicholson, D. (2018). Children Living with Domestic Violence: A Differential Response through Multi-agency Collaboration. *Australian Social Work, 71*(2), 162-174. doi:10.1080/0312407X.2017.1415366

Jackson, E. C., Renner, L. M., Flowers, N. I., Logeais, M. E., & Clark, C. J. (2020). Process evaluation of a systemic intervention to identify and support partner violence survivors in a multi-specialty health system. *BMC Health Services Research, 20*(1), 996. doi:10.1186/s12913-020-05809-y

Kaspiew, R., Maio, J. D., Deblaquiere, J., & Horsfall, B. (2012). *Evaluation of a pilot of legally assisted and supported family dispute resolution in family violence cases*. Retrieved from Australia https://apo.org.au/sites/default/files/resource-files/2012-12/apo-nid33293.pdf

Kimball, E., Rockhill, A., Heyen, C., & Keefe, S. H. (2018). The Safer Futures Model: Developing Partnerships between Intimate Partner Violence and Health Care Agencies. *Health and Social Work, 43*(3), 201-204. doi:10.1093/hsw/hly019

Lea, S. J., & Callaghan, L. (2016). "It Gave Me My Life Back": An evaluation of a specialist legal domestic abuse service. *Violence against Women, 22*(6), 704-721. doi:10.1177/1077801215610013

Macrae, R. (2014). The Caledonian system: An integrated approach to address men's domestic violence and improve the lives of women and children. *No To Violence Journal.*(Autumn), 37-58. Retrieved from https://library.nzfvc.org.nz/cgi-bin/koha/opac-detail.pl?biblionumber=4459

Macvean, M., Humphrey, C., Healey, L., Albers, B., Mildon, R., Connolly, M., . . . Spada-Rinaldis, S. (2015). *The PATRICIA Project: PAThways and research in collaborative inter-agency working: State of knowledge paper*. Retrieved from Sydney, NSW: https://anrowsdev.wpenginepowered.com/wp-content/uploads/2019/02/14_4.5-Landscapes-PATRICIA_F_0.pdf

Marjavi, A., Family Violence Prevention Fund, Ybanez, V., & Red Wind Consulting. (2010). *Building domestic violence health care responses in Indian Country: A promising practices report*. Retrieved from United States: http://ipvhealth.org/wp-content/uploads/2017/02/Promising-Practices-Report-Online-version.pdf

McArthur, M., & Thomson, L. (2011). Families' views on a coordinated family support service. *Family Matters*. Retrieved from https://aifs.gov.au/sites/default/files/fm89h_0.pdf

Meyer, S. (2014). Victims' experiences of short- and long-term safety and wellbeing: Findings from an examination of an integrated response to domestic violence. *Trends and Issues in Crime and Criminal Justice (478),* 1-7. Retrieved from https://www.aic.gov.au/publications/tandi/tandi478

Ministry of Justice. (2017). *Family violence risk assessment and management framework: A common approach to screening, assessing and managing risk*. Retrieved from New Zealand: https://www.justice.govt.nz/assets/Documents/Publications/family-violence-ramf.pdf

Ministry of Justice. (2019). *Sharing information safely: Summary of feedback: Guidance on sharing personal information under the Family Violence Act 2018*. Retrieved from New Zealand: https://www.justice.govt.nz/assets/Documents/Publications/Sharing-Information-Safely.pdf

Mossman, E., Paulin, J., & Wehipeihana, N. (2017). *Evaluation of the family violence Integrated Safety Response pilot: Final report*. Retrieved from https://thehub.swa.govt.nz/assets/documents/ISR_pilot_evaluation_FINAL.pdf

Mossman, E., Wehipeihana, N., & Bealing, M. (2019). *Evaluation of the family violence Integrated Safety Response pilot : Phase II - years 2 & 3: Final report*. Retrieved from Wellington, New Zealand https://www.justice.govt.nz/assets/Documents/Publications/nIG96VfM-ISR-Evaluation-Synthesis-Report.pdf

National Institute for Health Care Excellence. (2014). *Domestic violence and abuse: Multi-agency working*. Retrieved from UK: https://www.nice.org.uk/guidance/ph50

O'Leary, P., Young, A., Wilde, T., & Tsantefski, M. (2018). Interagency Working in Child Protection and Domestic Violence. *Australian Social Work, 71*(2), 175-188. doi:10.1080/0312407X.2017.1422773

O'Malley, R. (2013). CollaborACTION. *The No To Violence Journal., Ending Men's Violence Against Women and Children*, 51-71. Retrieved from https://library.nzfvc.org.nz/cgi-bin/koha/opac-detail.pl?biblionumber=4274

Pitt, K., Dheensa, S., Feder, G., Johnson, E., Man, M.-S., Roy, J., . . . Szilassy, E. (2020). Sharing reports about domestic violence and abuse with general practitioners: a qualitative interview study. *BMC Family Practice, 21*(1), 1-10. doi:10.1186/s12875-020-01171-4

Puccia, E., Redding, T. M., Brown, R. S., Gwynne, P. A., Hirsh, A. B., Hoffmann Frances, R. J., & Morrison, B. (2012). Using Community Outreach and Evidenced-Based Treatment to Address Domestic Violence Issues. *Social Work in Mental Health, 10*(2), 104-126. doi:10.1080/15332985.2011.601704

Robertson, N., & Payne, P. (2015). *A formative evaluation of the Waikato Family Safe Network Pilot*. Retrieved from Hamilton, New Zealand: https://hdl.handle.net/10289/9629

Ryan, J., & Block, M. (2020). *12 week review of cases referred to the family violence Integrated Safety Response (ISR): Review of 129 cases active with ISR from May to August 2018*. Retrieved from New Zealand https://www.police.govt.nz/sites/default/files/publications/isr-12-week-case-review-report.pdf

Smith, L. R., Gibbs, D., Wetterhall, S., Schnitzer, P. G., Farris, T., Crosby, A. E., & Leeb, R. T. (2011). Public health efforts to build a surveillance system for child maltreatment mortality: lessons learned for stakeholder engagement. *Journal of Public Health Management and Practice, 17*(6), 542-549. doi:10.1097/PHH.0b013e3182126b6b

Smith, N., & Harrell, S. (2011). *Forging new collaborations: A guide for rape crisis, domestic violence and disability organizations*. Retrieved from New York, USA: https://evawintl.org/wp-content/uploads/collaboration_report_020317_rev2.pdf

Spinney, A. (2012). *Home and safe? Policy and practice innovations to prevent women and children who have experienced domestic and family violence from becoming homeless*. Retrieved from Australia https://www.ahuri.edu.au/research/final-reports/196

Stanley, N., & Humphreys, C. (2014). Multi-agency risk assessment and management for children and families experiencing domestic violence. *Children and Youth Services Review, 47*(1), 78-85. doi:10.1016/j.childyouth.2014.06.003

Stanley, N., & Humphreys, C. (2017). Identifying the key components of a 'whole family' intervention for families experiencing domestic violence and abuse. *Journal of Gender-Based Violence, 1*(1), 99-115. doi:10.1332/239868017X14913081639164

Stanley, N., Miller, P., Foster, H. R., & Thomson, G. (2011). Children's Experiences of Domestic Violence: Developing an Integrated Response from Police and Child Protection Services. *Journal of Interpersonal Violence, 26*(12). doi:10.1177/0886260510383030

Steel, N., Blakeborough, L., & Nichola, S. (2011). *Supporting high-risk victims of domestic violence: A review of Multi-Agency Risk Assessment Conferences (MARACs)*. Retrieved from United Kingdom: https://www.bl.uk/collection-items/supporting-highrisk-victims-of-domestic-violence-a-review-of-multiagency-risk-assessment-conferences-maracs

Stevens, C., Ayer, L., Labriola, M., Faraji, S.-L., & Ebright, E. (2019). Detecting and reducing post-traumatic stress among children exposed to domestic violence: A multi-agency early intervention program. *Children & Youth Services Review, 101*, 261-269. doi:10.1016/j.childyouth.2019.03.055

Stewart, S. L. (2020). Enacting Entangled Practice: Interagency Collaboration in Domestic and Family Violence Work. *Violence against Women, 26*(2), 191-212. doi:10.1177/1077801219832125

Stylianou, A. M., & Ebright, E. (2021). Providing coordinated, immediate, trauma-focused, and interdisciplinary responses to children exposed to severe intimate partner violence: Assessing feasibility of a collaborative model. *Journal of Interpersonal Violence, 36*(5/6), NP2773-NP2799. doi:10.1177/0886260518769359

Tandon, D., Perry, D. F., Edwards, K., & Mendelson, T. (2020). Developing a model to address mental health, substance use, and intimate partner violence among home visiting clients. *Health Promotion Practice, 21*(2), 156-159. doi:10.1177/1524839919886293

Wehipeihana, N. (2019). *What's working for Māori? A Kaupapa Māori perspective on the responsiveness of the Integrated Safety Response pilot to Māori: Synthesis Evaluation Report*. Retrieved from Wellington, New Zealand: https://www.justice.govt.nz/assets/Documents/Publications/nOD858un-Kaupapa-Maori-Report.pdf

Wendt, S., Bastian, C., & Jones, M. (2021). Building Collaboration with Child Protection and Domestic and Family Violence Sectors: Trialling a Living Lab Approach. *British Journal of Social Work, 51*(2), 692-711. doi:10.1093/bjsw/bcaa206

Williams, V. N., Ayele, R., Shimasaki, S., Tung, G. J., & Olds, D. (2019). Risk assessment practices among home visiting nurses and child protection caseworkers in Colorado, United States: A qualitative investigation. *Health & Social Care in the Community, 27*(5), 1344-1352. doi:10.1111/hsc.12773

Wilson, D., & Webber, M. (2014). *The people's blueprint: transforming the way we deal with child abuse and domestic violence in New Zealand by [The Glenn Inquiry]*. Retrieved from New Zealand https://ndhadeliver.natlib.govt.nz/delivery/DeliveryManagerServlet?dps_pid=IE25595165&dps_custom_att_1=ilsdb

Wuest, J., Merritt-Gray, M., Dubé, N., Hodgins, M. J., Malcolm, J., Majerovich, J. A., . . . Varcoe, C. (2015). The Process, Outcomes, and Challenges of Feasibility Studies Conducted in Partnership with Stakeholders: A Health Intervention for Women Survivors of Intimate Partner Violence. *Research in Nursing and Health, 38*(1), 82-96. doi:10.1002/nur.21636

Zannettino, L., & McLaren, H. (2012). Domestic violence and child protection: Towards a collaborative approach across the two service sectors. *Child & Family Social Work, 19*(4), 421-431. doi.org/10.1111/cfs.12037
